# The precise molecular signals that control endothelial cell–cell adhesion within the vessel wall

**DOI:** 10.1042/BST20180377

**Published:** 2018-12-04

**Authors:** Lilian Schimmel, Emma Gordon

**Affiliations:** Institute for Molecular Bioscience, Genomics of Development and Disease Division, The University of Queensland, 306 Carmody Road, St Lucia, QLD 4072, Australia

**Keywords:** angiogenesis, cadherins, cell adhesion, endothelial cells, signalling

## Abstract

Endothelial cell–cell adhesion within the wall of the vasculature controls a range of physiological processes, such as growth, integrity and barrier function. The adhesive properties of endothelial cells are tightly controlled by a complex cascade of signals transmitted from the surrounding environment or from within the cells themselves, with the dynamic nature of cellular adhesion and the regulating signalling networks now beginning to be appreciated. Here, we summarise the current knowledge of the mechanisms controlling endothelial cell–cell adhesion in the developing and mature blood vasculature.

## Introduction

Blood vessels form complex branched networks consisting of arteries, capillaries and veins that supply oxygen and nutrients to all body tissues [1]. The early formation of the vascular network is achieved by a *de novo* assembly process termed vasculogenesis; however, the vast majority of vessels are formed through a process referred to as angiogenesis, where new vessels sprout from the pre-existing vasculature. As development progresses, vessels undergo active remodelling to ultimately form a stable, mature vessel network.

Vasculogenesis, angiogenesis and vascular remodelling are all highly dynamic processes, in which the adhesion of endothelial cells within the vessel wall is tightly controlled. This allows for cells to both grow and move past one another during sprouting, while maintaining a functional vascular barrier to prevent leakage from vessels. This highly dynamic and plastic nature of cell–cell adhesions is mediated through the junctional properties of the cells, primarily comprised of vascular endothelial cadherin (VE-cadherin)-based adherens junctions, and tight junctions primarily comprised of claudins, occludins, junctional adhesion molecules (JAMs), cingulin and nectins, many of which form homophilic complexes between endothelial cells and which connect junctions with the actin cytoskeleton [2–4]. The importance of junctions and cell adhesion during morphogenesis was demonstrated by early experiments where VE-cadherin was deleted in the mouse, resulting in embryonic lethality due to defects in vascular patterning [5,6].

The composition and localisation of adhesion molecules combined with the state of junctional tension transmitted to the actin cytoskeleton are central to cell–cell adhesion. These aspects are regulated through the extracellular cues presented to the cells and the intracellular signalling pathways which become activated. Vascular endothelial growth factor A (VEGF-A) was originally identified as a controller of cell–cell adhesion and vascular permeability [7]; however, the binding of VEGF-A to VEGF receptor 2 (VEGFR2) is also known to control endothelial cell identity, proliferation and migration [8,9]. A range of other signalling pathways act in consort with VEGF-A, and the ability of endothelial cells to integrate this wide range of signals to mediate physiological processes is critical for normal vessel function. Here, we review the signals controlling adherens junction-based cell–cell contacts in a range of vascular processes, such as vasculogenesis, angiogenesis, vascular leakage and leukocyte extravasation.

## Vessel formation

### Vasculogenesis

**Figure 1.**
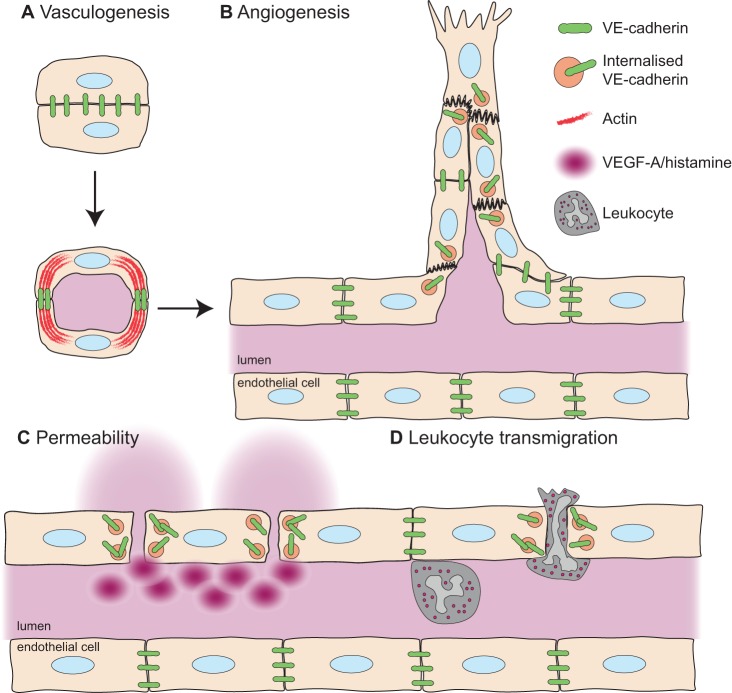
Adhesion within the vessel wall. Cell–cell adhesion plays a critical role in the development of the vascular system, regulating vasculogenesis (**A**), where the redistribution of adhesion from the apical surface of endothelial cells allows for opening of the lumen. Differential adhesion regulates the ability of angiogenic sprouts to elongate (**B**), with this adhesion tightly controlled to allow for the migration of cells within the sprout, while maintaining a non-leaky vasculature. During vascular permeability, cell adhesion is lost in response to permeability agents, such as VEGF-A, bradykinin or histamine (**C**), due to internalisation of junctional complexes. The transmigration of leukocytes across the vessel wall (**D**) is also tightly regulated by cell–cell adhesion, with both the breakdown and the sealing of junctions critical during extravasation.

Endothelial cell adhesion is a critical factor in the establishment of the primitive vascular plexus. During early vasculogenesis in the mouse, endothelial cell precursors arise at embryonic day 8 and subsequently assemble into an immature network of vascular cords. These precursors then differentiate to become lumenised, allowing for blood to pass through. This lumenisation of endothelial cells within the immature vascular network requires acquisition of cell polarity and disassembly of cell–cell adhesions to allow for opening of the vascular lumen (Figure 1A). This remodelling of the adhesions on the apical surface of the vasculature is dependent on a member of the Rho family of small GTPases, Cdc42, both *in vitro* [10] and *in vivo* [11]. The generation of active Cdc42 is regulated by Rasip1 (Ras-interacting protein 1), resulting in contractile nonmuscle myosin II (NMII) and F-actin pulling adhesion complexes away from the cell–cell contacts on the apical surface [12,13]. This is similar to what is observed during sprouting angiogenesis, where disruption of the interaction between actin and VE-cadherin results in a failure of lumen formation and maintenance [14]. Rasip1 is required for not only formation of the lumen during vasculogenesis, but also for the maintenance of the lumenised vasculature during development [15,16].

In addition to cytoskeletal rearrangements, VE-cadherin-mediated adherens junctions are critical for the remodelling and lumenisation of the primitive vascular plexus. During vascular remodelling, vessels in the embryonic yolk sac enlarge through a process of vascular fusion, where two existing vessels fuse to form one larger vessel with a common lumen [17,18]. This process of fusion is mediated by Notch signalling and blood flow-induced shear stress, with either Notch inhibition or reduced shear stress resulting in a hyperfused vascular plexus [18]. This process is, at least in part, mediated by VE-cadherin phosphorylation, which promotes its internalisation [19], leading to the rearrangement of adherens junction complexes and formation of a new vascular lumen. VE-cadherin can be phosphorylated or dephosphorylated by a range of kinases and phosphatases in response to biochemical signals [4] or via shear stress [19,20]. In the absence of a VE-cadherin phosphatase, vascular endothelial-protein tyrosine phosphatase (VE-PTP), mice exhibit an exceedingly hyperfused plexus due to increased VE-cadherin phosphorylation [21]. The hyperfusion can be reversed by inhibition of the non-receptor tyrosine kinase c-Src [22], an established promotor of VE-cadherin phosphorylation. Thus, VE-cadherin phosphorylation and adherens junction remodelling are essential during vessel fusion and lumen formation during vasculogenesis.

### Angiogenesis

Sprouting angiogenesis occurs by endothelial cells undergoing co-ordinated behaviour, involving a highly migratory, leading ‘tip cell’ and trailing ‘stalk cells’ [23–25]. The process of tip and stalk cell specification is mediated by a range of signals under the control of Notch, VEGF and Activin-like Kinase (ALK) receptors [26–30]. VEGFR2 activity is higher in tip cells, whereas Notch and ALK activation are induced in stalk cells through a feedback loop with VEGFR2 [25]. While tip and stalk cells are differentially specified by a range of signals, their behaviour is highly dynamic, with cells constantly interchanging positions, competing for the leading tip position [23,31–33]. However, as these cells jostle for position within the growing sprout, they still maintain contact with one another, indicating the requirement for rapid remodelling of cell–cell adhesions.

In order for cells to achieve a state of dynamic shuffling during sprouting, the importance of differential adhesion between heterogeneous endothelial cells in the growing sprout has been emphasised [32,34]. It is known that cell–cell adhesion is controlled by the junctional localisation of VE-cadherin (Figure 1B), whose internalisation is triggered by phosphorylation. In settings of low responsiveness to VEGF-A, VE-cadherin is neither phosphorylated nor internalised, leading to strongly adherent cells unable to migrate past each other and form elongated sprouts. Conversely, in settings of high VEGF-A where VE-cadherin is phosphorylated and internalised constantly, cells cannot maintain connections within the sprout and an aberrant, non-functional vasculature is formed [34].

**Figure 2.**
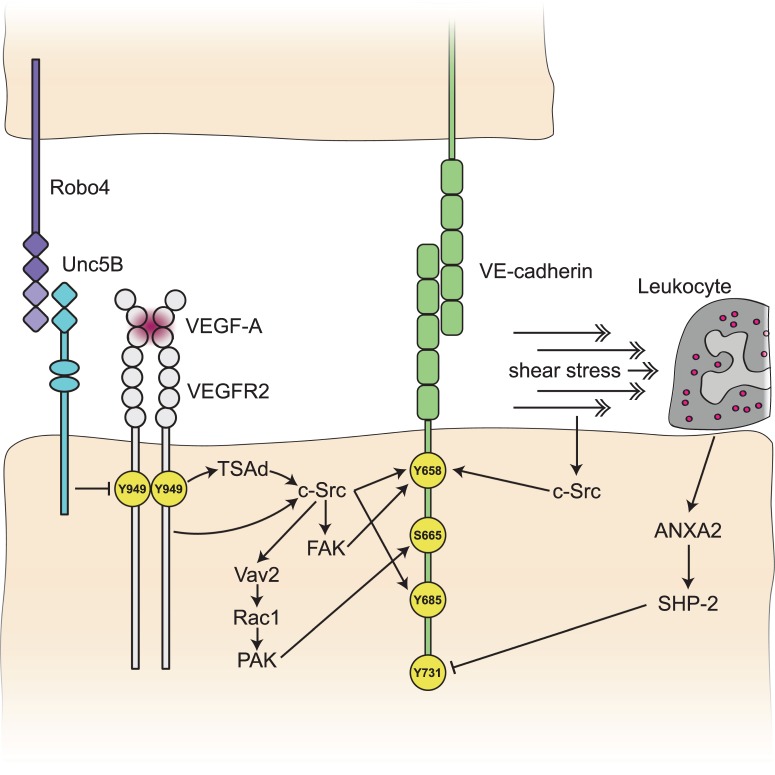
Signals guiding VE-cadherin phosphorylation in endothelial cells. Signalling pathways involved in regulating VE-cadherin phosphorylation and dephosphorylation on different tyrosine or serine residues. VEGFR2 activation results in VE-cadherin phosphorylation at its Tyr658, Ser665 or Tyr685 sites, mainly by c-Src activity. VEGFR2 activation at its Tyr949 site is regulated by Robo4 and Unc5B. c-Src-mediated Tyr658 phosphorylation is induced by shear stress. Leukocyte binding results in Tyr731 dephosphorylation via ANXA2 and the phosphatase SHP-2.

Exactly how VEGF-A downstream signals guide phosphorylation of VE-cadherin is not entirely clear. Phosphorylation of the tyrosine (Tyr) 949 residue in mouse VEGFR2 by VEGF-A mediates recruitment of the T-cell-specific adaptor (TSAd) protein, which is essential for activation of c-Src at cell–cell junctions [35]. While TSAd does not possess any intrinsic kinase activity, it acts as a ‘bridge’ to bind to and recruit c-Src to junctions. Once c-Src becomes activated at the junction, it phosphorylates VE-cadherin at two Tyr sites, 685 and 658, to mediate its internalisation (Figure 2) [36], which is sufficient to drive sprouting angiogenesis [37]. How VEGF-A/c-Src mediates differential adhesion in the growing sprout is unclear, but this process has been shown to be dependent on Notch. Cells with higher VEGFR2 activity and hence lower Notch activity (tip cells) have reduced junctional VE-cadherin, resulting in reduced cell adhesion and a greater migratory capacity [34]. The exact interplay between Notch and c-Src signalling is yet to be fully elucidated.

In addition to active remodelling of the cytoskeleton being critical during vasculogenesis, the correct polarisation of VE-cadherin and its coupling to the cytoskeleton is critical during angiogenic sprouting and anastomosis. Indeed, the VE-cadherin intracellular domain interacts with a range of intracellular partners, including β-catenin, plakoglobin and p120 [4], where these proteins can propagate intracellular signals and modulate interactions with the actin cytoskeleton. Additional information on the interaction with the cytoskeleton and VE-cadherin during angiogenesis is reviewed in detail by Szymborska and Gerhardt [3].

## Barrier function

### Vessel permeability

Cell–cell adhesion is critically important for the maintenance of vascular integrity and the prevention of leakage of fluid, proteins and cells from the bloodstream. Recent work has demonstrated that in addition to regulating sprouting angiogenesis, the VEGFR2-Tyr949/TSAd pathway controls vascular integrity in response to VEGF-A, but is not required for histamine-induced vascular leakage [35,36,38]. Similar to what is observed during sprouting angiogenesis [37], VEGF-A/VEGFR2-Tyr949/TSAd activation mediates c-Src recruitment to cell–cell junctions, where it phosphorylates VE-cadherin at its Tyr658 site, resulting in internalisation of VE-cadherin and junctional breakdown (Figure 1C). In mice with a mutation in the VEGFR2-Tyr949 site (tyrosine–phenylalanine) [38], phosphorylation of VE-cadherin is reduced and junctions maintain their integrity in pathological settings characterised by elevated VEGF-A, such as tumours. This not only results in decreased metastasis due to the inability of cancer cells to enter the bloodstream through paracellular gaps, but also to reduced leakage from mutant tumour vessels, and consequently, reduced oedema in the primary tumour, increasing the efficiency of chemotherapy. The degree of activation of VEGFR2-Tyr949 can be controlled by Robo4, a transmembrane protein which activates the endothelial receptor UNC5B, that is known to be critical for endothelial barrier function [39,40]. Deletion of Robo4 results in increased VEGFR2-Tyr949 activation, increased c-Src activation and increased vascular leakage in response to VEGF-A (Figure 2) [41], presumably due to increased VE-cadherin phosphorylation. Exactly how Robo4-UNC5B restricts VEGFR2-Tyr949 phosphorylation remains to be determined, but may be through docking sites on UNC5B for a VEGFR2-Tyr949 phosphatase.

In addition to the VE-cadherin Tyr658 site, Tyr685 has been shown to be critical for endothelial permeability [42] and can be phosphorylated by c-Src in response to VEGF-A [43]. In addition to tyrosine phosphorylation, VE-cadherin can be phosphorylated on its Serine (Ser)665 residue in a Src-Vav2-Rac-p21-activated kinase (PAK) dependent manner downstream of VEGFR2 activation (Figure 2) [44]. This phosphorylation at Ser665 results in β-arrestin2 binding, internalisation into clathrin-coated early endosomes, and subsequent disruption of cell–cell junctions. The exact contribution of each of VE-cadherin's phosphorylation sites to adherens junction morphology is yet to be completely understood. However, dissociation of VE-PTP from VE-cadherin is essential for VEGF-A-induced vascular leakage [45,46], revealing that VE-cadherin phosphorylation is essential for the opening of cell junctions and vascular permeability.

Similar to what is observed during vasculogenesis [22], phosphorylation of VE-cadherin can be mediated by hemodynamic forces during vascular permeability. Arteries and veins possess intrinsically different cell–cell adhesion within the vessel wall, resulting in different sensitivities to vascular leakage/permeability. For example, veins are more sensitive to inflammation-induced leakage than adjacent arteries [47]. Orsenigo et al. [19] found that VE-cadherin is constantly phosphorylated at its Tyr658 and Tyr685 residues in veins but not in arteries, which is mediated by shear stress-induced c-Src activity at cell–cell junctions. Tyr658 and Tyr685 basal phosphorylation does not result in increased leakage from veins under normal states and activation of c-Src alone is not sufficient to induce breakdown of endothelial cell monolayer integrity [48]. However, when treated with permeability-inducing agents bradykinin or histamine, phosphorylated VE-cadherin on veins becomes internalised and degraded through the ubiquitin pathway [19]. This suggests that VE-cadherin phosphorylation by c-Src is essential, but not sufficient, to induce vascular permeability. The exact contribution of VE-cadherin to vascular leakage is further clouded by findings that postnatal deletion of VE-cadherin results in junctional instability only in organs exposed to high shear stress, such as lung and heart [49]. It is likely that other structures, such as the cytoskeleton or surrounding basement membrane, act to reinforce junctions in established blood vessels.

In addition to c-Src, there are many other kinases that can mediate VE-cadherin internalisation (reviewed in ref. [4]). One of interest, focal adhesion kinase (FAK), can bind to VE-cadherin and phosphorylate β-catenin, resulting in dismantling of adherens junctions and increased vascular permeability in response to VEGF-A [50]. FAK can also directly phosphorylate VE-cadherin on its Tyr658 residue, promoting vascular permeability and tumour metastasis [51], suggesting an interplay between both adherens junctions and focal adhesion complexes.

While VEGF is a potent permeability agent, other permeability-inducing factors such as inflammatory cytokines (bradykinin and histamine) and growth factors [including Sphingosine-1-phosphate (S1P), Angiopoietin (Ang)-1 and transforming growth factor β1 (TGFβ1)] are efficient activators of vascular permeability. Furthermore, in addition to paracellular transport discussed here, transcellular pathways involving caveolae, vesiculo-vacuolar organelles (VVOs), and fenestrae can mediate vascular permeability. Additional information can be found in detailed reviews on vascular permeability from Claesson-Welsh [52] and Park-Windhol and D'Amore [53].

### Leukocyte transmigration

During extravasation of leukocytes, endothelial cells need to open up their cell–cell junctions in order to allow for passage of cells from the bloodstream to the surrounding tissue (Figure 1D). The importance of VE-cadherin internalisation in this process was demonstrated by the generation of a mouse with a VE-cadherin–α-catenin fusion construct that induces strong and stabilised endothelial junctions. Leukocyte extravasation is almost completely inhibited in these mice and they are resistant to VEGF-A or histamine-induced vascular leakage, indicating the importance of dynamic VE-cadherin adhesions at endothelial cell junctions [54].

The opening of endothelial cell junctions during leukocyte extravasation or during increased vessel permeability are two separate processes, each regulated by different signalling pathways. As mentioned above, phosphorylation of VE-cadherin at Tyr658 and/or Tyr685 by c-Src upon VEGF-A/histamine stimulation results in VE-cadherin internalisation and subsequent increased vessel permeability [38,42,43,45]. However, during leukocyte extravasation, the dephosphorylation of yet another Tyr phosphorylation site in VE-cadherin, Tyr731, is triggered by binding of leukocytes. A Tyr731 mutation (tyrosine–phenylalanine) of VE-cadherin showed reduced rates of leukocyte extravasation both *in vitro* and *in vivo*, while VEGF-A-induced permeability was not affected in these mice [42,55]. Tyr731 dephosphorylation occurs via the phospholipid-binding protein, annexin A2 (ANXA2), which supports the assembly of tyrosine phosphatases, including Src Homology Phosphatase 2 (SHP-2), at the cytoplasmic tail of VE-cadherin [56]. Dephosphorylation of VE-cadherin Tyr731 by SHP-2 leads to binding of the adaptin complex AP-2, which serves as a clathrin adaptor and causes VE-cadherin endocytosis, opening of endothelial cell–cell junctions and leukocyte passage. Depletion of either ANXA2 or SHP-2 induces phosphorylation of VE-cadherin Tyr731 and inhibition of leukocyte extravasation (Figure 2) [42,56].

After the passage of leukocytes, it is essential to restore the VE-cadherin-based cell–cell junctions in order to maintain vascular barrier function. Following leukocyte transmigration, GTPase Rac1 is recruited to the cell junctions, mediated by another small GTPase RhoB, where subsequent junctional Rac1 activation acts to close the endothelial junction gaps by ventral lamellipodia formation [57,58]. Downstream of Rac1, activation of the actin nucleator ARP2/3 induces actin cytoskeleton remodelling and formation of lamellipodia that reconnect to the neighbouring endothelial cell. The induced lamellipodia not only protrudes to the neighbouring cell, but is also involved in the formation of new VE-cadherin adhesion sites in order to restore the junction [59,60]. These junction-based lamellipodia are also critical for cell rearrangements during angiogenesis [61,62], revealing that endothelial cell–cell adhesion mechanisms may be conserved across physiological processes.

## Summary

Cell–cell adhesion is a critical part of the formation, maintenance and function of the blood vasculature. VE-cadherin, a key component of adherens junctions, can be regulated by a range of factors, such as kinases, phosphatases and hemodynamic forces, all of which guide its stability at the cell junction. The exact signalling cascades which are active at cell–cell junctions are under exquisite spatiotemporal control, and we are only beginning to unravel the specific cues guiding these pathways. An understanding of this signalling and the subsequent adhesive properties of VE-cadherin at junctions will be of great interest, as during disease the strengthening or loosening of cell junctions may be beneficial or detrimental, depending on the physiological need. Elucidating the mechanisms of vascular cell adhesion in response to different physiological cues will allow us to obtain more tools to ultimately manipulate the plasticity of the vasculature during disease.

## Perspectives

- The regulation of cell–cell adhesion is critical during blood vessel formation and function, as cells within the vessel wall need to be able to move around each other while maintaining a functional vascular barrier.
- Cellular adhesion in the vessel wall is largely controlled by VE-cadherin-mediated adherens junctions. VE-cadherin can be internalised by phosphorylation, resulting in reduced junctional strength and cell adhesion. Exactly how the exquisite regulation of VE-cadherin phosphorylation and internalisation is controlled remains unclear.
- Future studies will unravel how and why specific sites of phosphorylation within VE-cadherin controls cell adhesion in different physiological conditions.

## Abbreviations

ALK: Activin-like Kinase
ANXA2: annexin A2
FAK: focal adhesion kinase
Rasip1: Ras-interacting protein 1
Ser: serine
SHP-2: Src Homology Phosphatase 2
TSAd: T-cell-specific adaptor
Tyr: tyrosine
VE-cadherin: vascular endothelial cadherin
VEGF-A: vascular endothelial growth factor A
VEGFR2: VEGF receptor 2
VE-PTP: vascular endothelial-protein tyrosine phosphatase
